# Comparison of the diagnostic performance and impact on management of 18F-FDG PET/CT and whole-body MRI in multiple myeloma

**DOI:** 10.1007/s00259-020-05182-2

**Published:** 2021-01-19

**Authors:** Olwen Westerland, Ashik Amlani, Christian Kelly-Morland, Michal Fraczek, Katherine Bailey, Mary Gleeson, Inas El-Najjar, Matthew Streetly, Paul Bassett, Gary J. R. Cook, Vicky Goh, on behalf of the Myeloma Imaging Research Group at Guy’s & St Thomas’ Hospital, London and King’s College London, Joanna Bell, Isabel Dregely, Adrian Green, Renyang Gu, Ulrike Haberland, Sami Jeljeli, Majid Kazmi, Nessa Muhidun, Sarah Natas, Radhouene Neji, Francesco Padormo, John Spence, J. James Stirling, Manil Subesinghe, Hema Verma, Zaid Viney

**Affiliations:** 1 Department of Cancer Imaging, School of Biomedical Engineering and Imaging Sciences, King’s College London, St Thomas’ Hospital, London, UK; 2 Clinical Imaging and Medical Physics, Guy’s and St Thomas’ NHS Foundation Trust, London, UK; 3 Haematology and Oncology, Guy’s and St Thomas’ NHS Foundation Trust, London, UK; 4 Statsconsultancy Ltd, Amersham, UK; 5 King’s College London and Guy’s and St Thomas’ PET Centre, St Thomas’ Hospital, London, UK

**Keywords:** Myeloma, 18F-fluorodeoxyglucose positron emission tomography computed tomography, Whole-body magnetic resonance imaging, Diagnosis

## Abstract

**Purpose:**

Comparative data on the impact of imaging on management is lacking for multiple myeloma. This study compared the diagnostic performance and impact on management of 18F-fluorodeoxyglucose positron emission tomography/computed tomography (18F-FDG PET/CT) and whole-body magnetic resonance imaging (WBMRI) in treatment-naive myeloma.

**Methods:**

Forty-six patients undergoing 18F-FDG PET/CT and WBMRI were reviewed by a nuclear medicine physician and radiologist, respectively, for the presence of myeloma bone disease. Blinded clinical and imaging data were reviewed by two haematologists in consensus and management recorded following clinical data ± 18F-FDG PET/CT or WBMRI. Bone disease was defined using International Myeloma Working Group (IMWG) criteria and a clinical reference standard. Per-patient sensitivity for lesion detection was established. McNemar test compared management based on clinical assessment ± 18F-FDG PET/CT or WBMRI.

**Results:**

Sensitivity for bone lesions was 69.6% (32/46) for 18F-FDG PET/CT (54.3% (25/46) for PET component alone) and 91.3% (42/46) for WBMRI. 27/46 (58.7%) of cases were concordant. In 19/46 patients (41.3%) WBMRI detected more focal bone lesions than 18F-FDG PET/CT. Based on clinical data alone, 32/46 (69.6%) patients would have been treated. Addition of 18F-FDG PET/CT to clinical data increased this to 40/46 (87.0%) patients (*p* = 0.02); and WBMRI to clinical data to 43/46 (93.5%) patients (*p* = 0.002). The difference in treatment decisions was not statistically significant between 18F-FDG PET/CT and WBMRI (*p* = 0.08).

**Conclusion:**

Compared to 18F-FDG PET/CT, WBMRI had a higher per patient sensitivity for bone disease. However, treatment decisions were not statistically different and either modality would be appropriate in initial staging, depending on local availability and expertise.

**Supplementary Information:**

The online version contains supplementary material available at 10.1007/s00259-020-05182-2.

## Introduction

Advanced imaging with whole-body computed tomography (WBCT), whole-body magnetic resonance imaging (WBMRI), or 18F-fluorodeoxyglucose positron emission tomography/computed tomography (18F-FDG PET/CT) is now recommended by the International Myeloma Working Group (IMWG) for skeletal assessment of myeloma due to their superior performance over radiographic skeletal survey [1]. The IMWG consensus guidelines remain pragmatic, recognising that the imaging modality choice is often influenced by local availability, expertise, and cost. Nevertheless, it is important that the selected baseline imaging test is sufficiently sensitive to detect small volume disease with high specificity and impacts on patient management.

A small number of studies, with limited sample sizes, have compared the diagnostic accuracy of 18F-FDG PET/CT and WBMRI in myeloma [2–5], but to date, there has been a lack of comparative data of the impact on management of 18F-FDG PET/CT and WBMRI. Both techniques have advantages. While WBMRI has superior sensitivity for diffuse bone marrow infiltration [6], 18F-FDG PET/CT better demonstrates lesion viability following treatment [7]; both convey prognostic information [8, 9]. Additionally, in patients with smouldering myeloma, the presence of >1 focal bone lesion detected by WBMRI [10] or the presence of a focal FDG-avid lesion [11] is a strong prognostic factor for progression to myeloma such that they are now recognised as an indication to treat otherwise asymptomatic myeloma [12, 13].

We hypothesised that the greater sensitivity of WBMRI for detection of myeloma compared to 18F-FDG PET/CT would alter patient management in a greater proportion of cases. Thus, the primary aim of this study was to compare the diagnostic performance of 18F-FDG PET/CT and WBMRI for bone disease in patients with a new myeloma diagnosis and to assess whether management differed depending on which imaging was performed initially. We also assessed interobserver agreement of 18F-FDG PET/CT and WBMRI for bone disease detection in a subset of patients.

## Methods

This retrospective study was granted institutional approval by our Institutional Service Evaluation committee and the requirement for informed consent for data use was waived.

### Patients

Consecutive patients referred to our centre with a suspected or confirmed new diagnosis of myeloma that underwent 18F-FDG PET/CT and WBMRI between March 2014 and May 2018, prior to treatment, were included. Patients were excluded if they did not fulfil inclusion criteria.

### Imaging

#### 18F-FDG PET/CT

Following a 6-h fast, up to 400 MBq 18F-FDG was administered intravenously if blood glucose was < 10 mmol/L. At 60 +/− 5 min post-injection, imaging was acquired from skull vertex to feet at 3.5 min per bed position with an axial field of view of 15.7 cm and an 11-slice overlap between bed positions, using a GE Discovery 710 PET-CT scanner (GE Healthcare, Chicago, US). A low-dose CT scan (140 kV, mA 15-100, noise index 40, 0.5 s rotation time, and 40 mm collimation) was performed at the start of imaging to provide attenuation correction and an anatomical reference. PET image reconstruction included scanner-based corrections for radiotracer decay, scatter, randoms, and dead-time. Emission sinograms were reconstructed with an ordered subset expectation maximisation algorithm (2 iterations, 24 subsets).

#### WBMRI

WBMRI consisting of T2-weighted fast-spin echo, pre-and post-contrast agent T1-weighted Dixon spoiled 3D gradient-recalled echo and diffusion-weighted echo-planar imaging (with *b* value = 50 and 900 s/mm^2^) sequences from the skull vertex to below knees was performed at 1.5 T (Magnetom Aera, Siemens Healthcare, Erlangen, Germany) (Supplemental Table 1). WBMRI study duration was approximately 45 min, depending on patient height.

#### Image analysis

18F-FDG PET/CT and WBMRI imaging were reviewed independently by an experienced nuclear medicine physician and radiologist, respectively specialising in whole body and myeloma imaging. The presence/absence of focal and/or diffuse disease and number of focal lesions were recorded. Where focal lesions were present, their number was recorded as follows: <5; 5 to 10; or >10. Focal bone lesions were categorised as 18F-FDG-positive, i.e. standardised uptake value (SUV) greater than background bone marrow, or 18-FDG-negative. The presence or absence of an osteolytic lesion typical of myeloma on the CT component using bone window settings was also recorded, whether 18F-FDG-avid or not. For the purposes of qualitative assessment for diffuse marrow infiltration on 18F-FDG PET/CT, the background bone marrow was considered positive if bone marrow 18F-FDG uptake exceeded hepatic activity (bone marrow SUVmax: hepatic SUVmax ratio >1.0) [14]. Bone marrow involvement at WBMRI was recorded as follows: normal, focal, diffuse, salt and pepper or a combination of abnormal patterns [15].

Further evaluation was performed in a subset of patients (25%) by 2 additional readers (a nuclear medicine physician and radiologist, respectively) to assess inter-observer agreement. For WBMRI, a total lesion score was assigned to facilitate assessment of inter-reader agreement of number of lesions across the different sites. The number of bone lesions were scored as follows: score 0=0 lesions; 1=1-4 lesions; 2=5-10 lesions; 3 =>10 lesions; for following 7 skeletal regions: skull, cervical spine, thoracic spine, lumbar spine, pelvis, long bones, ribs/other.

#### Clinical data collection

Electronic patient records were reviewed and the following patient data were collected and anonymised; demographic data, serum haemoglobin, creatinine, free light chain ratio, paraprotein level and bone marrow trephine plasma cell percentage at diagnosis; final recorded diagnosis following multidisciplinary consensus clinical discussion and international myeloma stage (ISS) served as the reference standard for presence of disease status.

#### Management assignment

Anonymised clinical and imaging data were reviewed by two consultant haematologists in consensus. Imaging data included; study positive or negative for disease by IMWG criteria, number of focal bone lesions (and for 18F-FDG PET/CT, whether bone lesions were FDG positive or negative, and with/without associated lytic component on CT), WBMRI disease pattern and likely presence/absence of diffuse infiltration on 18F-FDG PET/CT based on objective assessment of background bone marrow avidity compared to the liver. Clinical data alone, clinical and 18F-FDG PET/CT data and clinical and WBMRI data were reviewed separately and virtual management decisions made (either treat as myeloma or active surveillance) as per institutional treatment guidelines . Data was randomised and reviewed on separate occasions, with an interval of at least 1 week, to minimise recall bias.

#### Reference standard

Concordance and diagnostic performance of WBMRI and 18F-FDG PET/CT were assessed against the IMWG definition of myeloma bone disease which states that there should be one or more 5 mm osteolytic bone lesions at CT or 18F-FDG PET/CT and more than one unequivocal bone marrow lesion (measuring at least 5 mm) at WBMRI [13] and clinical reference standard for disease status, including bone marrow biopsy and final recorded diagnosis following multidisciplinary consensus clinical discussion.

### Statistical analysis

Sensitivity of bone marrow SUVmax: hepatic SUVmax ratio >1.0 for detection of bone marrow infiltration (where bone marrow trephine plasma cell percentage of >10% was taken as the reference standard) was assessed. Spearman rank correlation was performed for assessment of correlation between background bone marrow avidity, SUVmax at the iliac crest, and bone marrow plasma cell percentage; and WMBRI bone marrow infiltration pattern and bone marrow plasma cell percentage. Inter-reader agreement for disease scoring by 18F-FDG PET/CT or WB-MRI was assessed using Cohen’s Kappa for categorical and Intraclass Correlation Coefficient for continuous data. The McNemar test was used to assess the significance of the difference between the proportion of different management decisions (either active treatment or surveillance) based on (i) clinical information alone compared with decision based on (ii) clinical information + 18F-FDG PET/CT result, or (iii) clinical information + WBMRI result, and finally (iv) comparing decision based on clinical information + 18F-FDG PET/CT and clinical information + WBMRI. *P*<0.05 denoted statistical significance.

## Results

### Patients

Forty-six patients (24 male, median age 63 years, range: 36-86 years) with a suspected or confirmed new clinical diagnosis of myeloma received both pre-treatment WBMRI and 18F-FDG PET/CT between March 2014 and May 2018. The median time interval between imaging studies was 7 days (range: 0-56 days). 89.1% (41/46 patients) had symptomatic myeloma and 6.5% (3/46 patients) had smouldering myeloma. 4.3% (2/46 patients) had multifocal plasmacytoma, with diagnosis upgraded to myeloma. Median serum paraprotein was 29.5 g/L (range: 2–77 g/L). All patients underwent baseline bone marrow trephine and biopsy. Median bone marrow plasma cell percentage was 30% (range: 1-90%). Clinical characteristics are summarised in Table 1.

**Table 1:** Summary of clinical characteristics

Clinical characteristics	
No. of patients	46
Sex	
Male	24
Female	22
Age	
Median (range), years	63 (36-86)
Diagnosis	
Symptomatic myeloma	41
Smouldering myeloma	3
Multiple plasmacytoma variant	2
International staging system (ISS)	
ISS 1	19
ISS 2	17
ISS 3	10
Bone marrow plasma cell percentage	
Median (range), %	30 (1-90%)
Serum haemoglobin level	
Median (range), g/L	106 (74-138)
Serum creatinine level	
Median (range), umol/L	85.5 (45-585)
Serum calcium level	
Median (range), mmol/L	2.45 (2.16-3.90)
Serum paraprotein level	
Median (range), g/L	29.5 (2-77)

### Detection of focal lesions

The per patient sensitivity of WBMRI for detecting myeloma bone disease was 91.3% (42/46 patients) compared with 69.6% (32/46 patients) for 18F-FDG PET/CT, (69.6% (32/46 patients) for the CT component alone and 54.3% (25/46 patients) for the PET component alone for the detection of bone lesions). There were no cases where 18F-FDG PET/CT was positive for disease detection and WBMRI was negative.

**Fig. 1:**
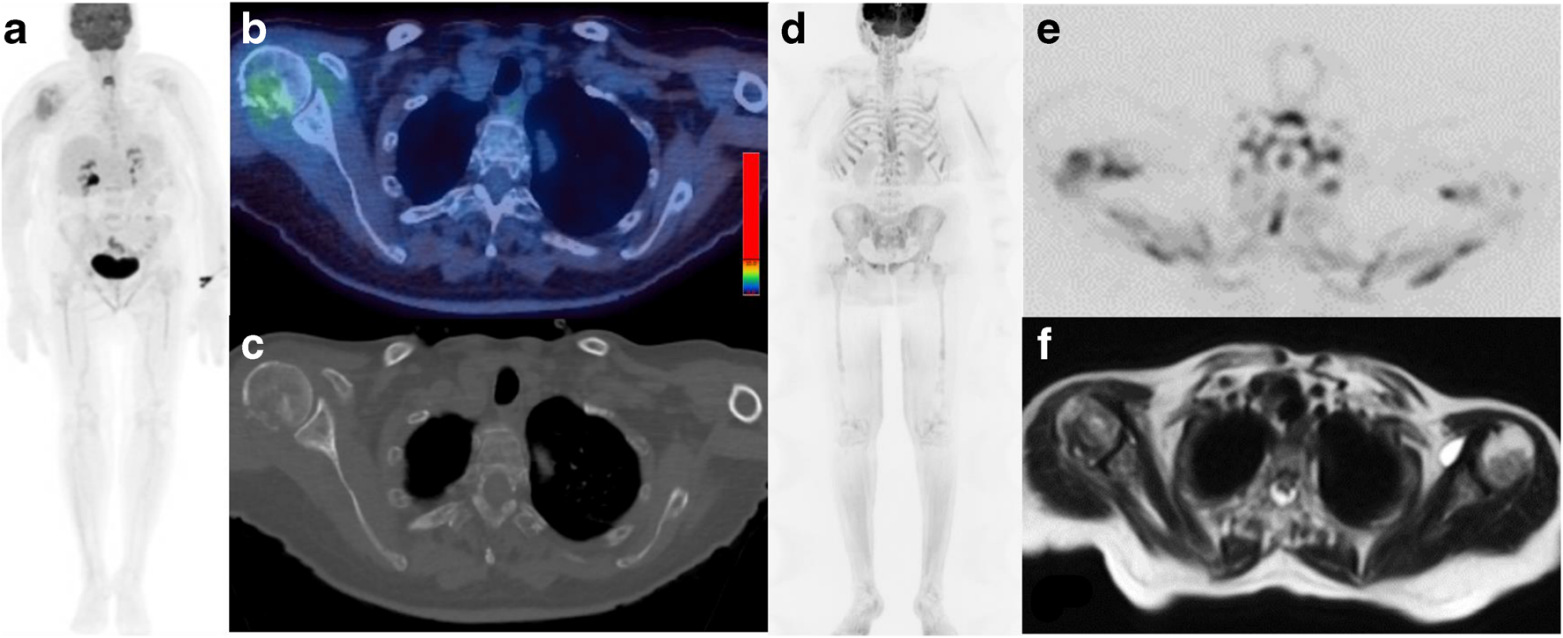
Seventy-six-year-old female with symptomatic myeloma and 80% bone marrow infiltration. 18F-FDG PET/CT (a: Whole body MIP; b: Axial fused image; c: Axial CT image (bone windows)) and WBMRI (d: b900 inverted whole body MIP; e: Axial inverted b900 image; f: Axial T2-weighted image) demonstrates a pathological fracture of the right humeral head as well as more widespread disease with multiple focal lesions (>10) and on both the CT component of the 18F-FDG PET/CT and WBMRI. Beyond the FDG uptake at the fracture site, the background bone marrow: liver FDG uptake ratio was <1.0

**Fig. 2:**
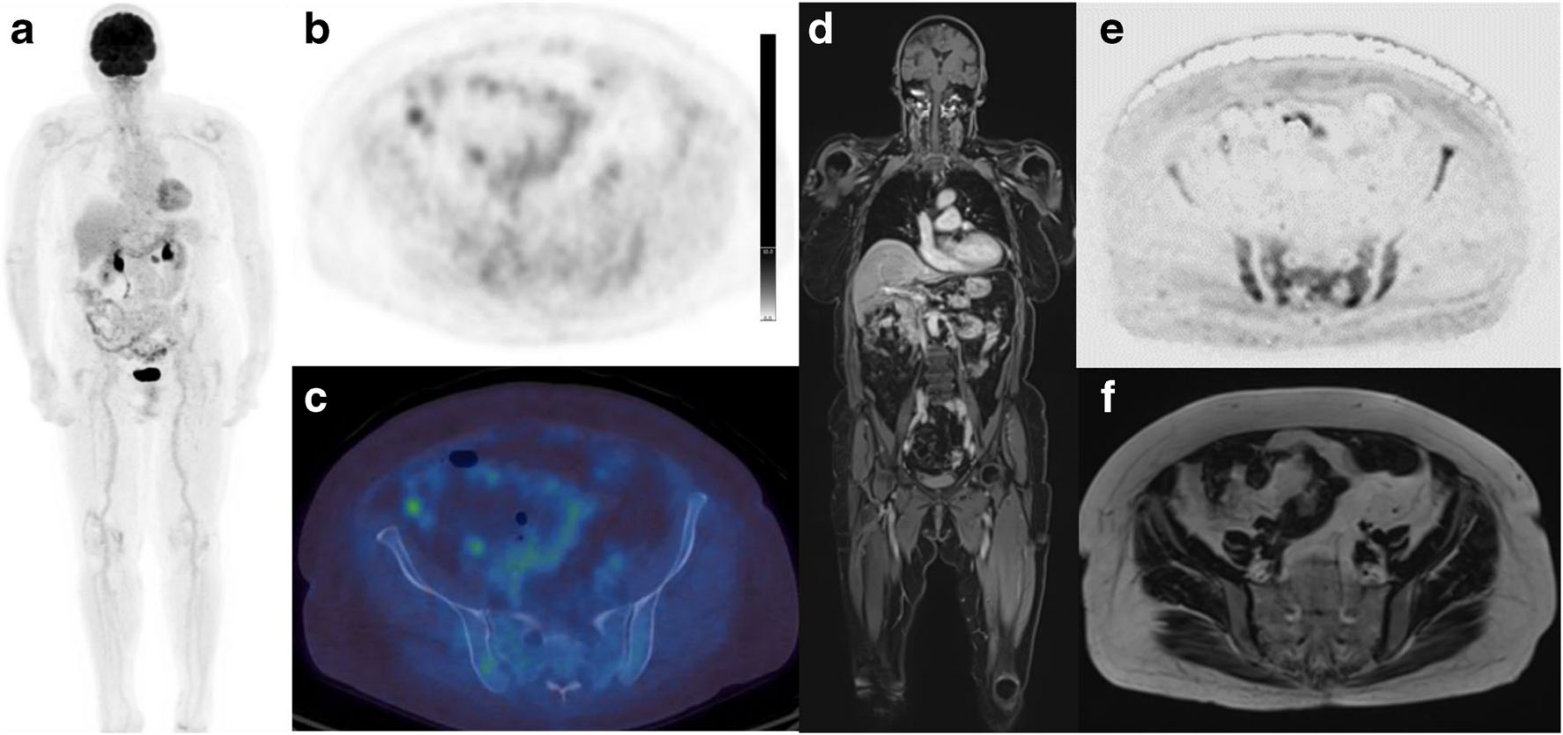
Eighty-three-year-old female with smouldering myeloma, rising serum paraprotein and 15% bone marrow infiltration. 18F-FDG PET/CT (a: Whole body MIP; b: Axial PET image; c: Axial fused image) and WBMRI (d: Coronal contrast enhanced T1-weighted fat-suppressed Dixon image; e: Axial inverted diffusion b900 image; f: Axial T2-weighted image) are shown. Pelvic focal lesions and marrow infiltration seen on the diffusion and contrast enhanced T1-weighted images are not as easily detected on 18F-FDG PET/CT (bone marrow: liver FDG uptake ratio <1.0)

In 58.7% (27/46) patients, WBMRI and the CT component of 18F-FDG PET/CT detected a concordant number of focal bone lesions whilst in 41.3% (19/46) WBMRI detected a greater number of focal bone lesions (Table 2; Fig. 1). Of note, in 10/19 discordant patients with negative 18F-FDG PET/CT studies, there were more than 10 lesions present on the WBMRI. In the 3 patients with smouldering myeloma, all had negative 18F-FDG PET/CT studies whilst 2/3 (66.6%) also had negative WBMRI studies. One patient with smouldering myeloma had focal bone lesions on WBMRI and their diagnosis was upgraded to myeloma (Fig. 2). In patients with 18F-FDG PET/CT positive disease (PET ± CT, 32/46 (69.9%), 23/32 (71.8%) patients had both FDG-avid and CT lytic lesions totalling 69 lesions, while 13/32 (40.6%) patients had FDG-avid lesions with no accompanying lytic component totalling 83 lesions.

**Table 2:** Comparison of number of focal lesion detected for 18F-FDG PET/CT and WBMRI

Imaging	Number of lesions			
	0	<5	5–10	>10
All cases, *n* = 46				
18F-FDG PET/CT	15	11	1	19
WBMRI	5	5	2	34
Concordant cases, *n* = 27				
WBMRI = 18F-FDG PET/CT	5	3	0	19
Discordant cases, *n* = 19				
WBMRI where 18F-FDG PET/CT = 0	-	2	-	10
WBMRI where 18F-FDG PET/CT <5	-	-	2	6
WBMRI where 18F-FDG PET/CT				
5-10	-	-	-	1

### Assessment of marrow involvement

In 25/46 (54.3%) of patients, bone marrow 18F-FDG SUVmax exceeded hepatic SUVmax. There was a weak positive correlation between SUVmax at the right iliac crest and bone marrow plasma cell percentage (*r* = 0.311, *p* = 0.036); the sensitivity of bone marrow SUVmax: hepatic SUVmax ratio >1.0 for detection of bone marrow involvement was 36.4%, where bone marrow involvement was defined as bone marrow trephine plasma cell percentage of > 10%. The two patients with multifocal plasmacytoma were excluded from this analysis.

WBMRI infiltration patterns in imaging positive studies were as follows: normal = 4/46 (8.7%), focal = 15/46 (32.6%) and salt and pepper/diffuse (alone or in combination with focal pattern) = 27/46 (58.7%). There was no significant correlation between WBMRI infiltration pattern and bone marrow plasma cell percentage (*r* = 0.193) (*p* = 0.2)

### Inter-reader agreement

Inter-reader agreement was assessed in a subset of 12/46 (26.1%) patients.

#### 18F-FDG PET/CT

There was moderate agreement between the two readers in scoring for the presence/absence of focal CT and PET disease, with a kappa score of 0.44 and 0.47, respectively. Identification of CT disease was discordant in 3/12 patients, where CT positive but FDG-negative lesions were noted by one reader. Identification of FDG-positive disease was also discordant in 3/12 patients.

#### Whole-body MRI

There was excellent agreement between the two readers in scoring for the presence/absence of focal lesions on a per patient basis (kappa score, 1.00). With respect to the scoring of the number of lesions across the 7 regions, mean ± SD focal lesion score was 7.4 ± 5.7 for Reader 1 and 7.7 ± 5.5 for Reader 2; with an intraclass correlation coefficient of 0.95 [95%CI: 0.84-0.99]. With respect to the presence/absence of bone marrow infiltration, there was good agreement between both readers with a kappa score of 0.74; one patient with a bone marrow infiltration percentage of 90% on biopsy was assigned incorrectly as negative by one reader.

### Impact on clinical management

Based on the review of clinical data alone, 32/46 (69.6%) of patients would be treated for myeloma rather than active surveillance, in accordance with institutional treatment protocols. Review of clinical data with 18F-FDG PET/CT resulted in treatment of 40/46 (87.0%) patients, and review of clinical data with WBMRI resulted in treatment of 43/46 (93.5%) patients.

Of the 3/46 (6.5%) cases where review of clinical and WBMRI data resulted in surveillance rather than treatment, both WBMRI and 18F-FDG PET/CT were negative for bone disease by IMWG criteria. Of the further 3 cases where review of clinical and 18F-FDG PET/CT data resulted in surveillance, 18F-FDG PET/CT was negative by IMWG criteria in all cases (2 cases PET component positive, CT component negative) whilst WBMRI demonstrated focal disease in all cases.

14/46 (30.4%) patients had a negative 18F-FDG PET/CT by IMWG criteria. However, 6/14 (42.9%) would have been treated on the basis of clinical data alone. Using the McNemar test, significance of differences between percentage treated was *p* = 0.02 for clinical data alone compared with clinical data + FDG-PET/CT result, *p* = 0.002 for clinical data compared with clinical data + WBMRI result and *p* = 0.08 for clinical data + FDG-PET/CT result compared with clinical data + WBMRI result (Table 3).

**Table 3:** Comparison of clinical management with clinical data only; clinical data + 18F-FDG PET/CT; and clinical data + WBMRI

Method	% treated	% difference (95% CI)	*P* value
Clinical data only	70%	0	0.02
Clinical data + PET/CT	87%	17% (1%, 33%)	
Clinical data only	70%	0	0.002
Clinical data + WBMR	93%	24% (8%, 40%)	
Clinical data + PET/CT	87%	0	0.08
Clinical data + WBMR	93%	7% (-3%, 16%)	

## Discussion

Our study confirms that WBMRI detects skeletal disease in a higher number of treatment-naïve patients than 18F-FDG PET/CT and also detects a higher number of lesions per patient with concordance in disease positivity/lesion number in only 59% of patients. Just under half of patients (45.7%) did not have focal or diffuse FDG avid disease in our cohort. Observer agreement was moderate to substantial for 18F-FDG PET/CT and WBMRI, respectively.

Imaging with either 18F-FDG PET/CT or WBMRI resulted in a change in management in up to 23.9% (11/46) of patients, where review of clinical data alone would have resulted in surveillance rather than treatment. WBMRI resulted in a decision to treat in an additional 6.5% (3/46) of patients compared with 18F-FDG PET/CT. In cases of a negative 18F-FDG PET/CT scan, a negative impact on management (i.e. surveillance versus treatment) was mitigated by positive clinical data.

No previous study has assessed management change, although, there have been a limited number of studies comparing diagnostic performance of WBMRI and 18F-FDG PET/CT in myeloma [3–5, 16–19], most with small patient numbers and heterogeneous study methodology [2]. One prospective study comparing WBMRI and 18F-FDG PET/CT pre and post-treatment in 56 patients [5], found that WBMRI had a greater sensitivity than 18F-FDG PET/CT in detection of bone lesions before treatment (WBMRI sensitivity = 94%, 18F-FDG PET/CT sensitivity = 75%, p=0.0039). Nevertheless, 18F-FDG PET/CT had a greater specificity in detection of residual disease post-treatment, with equal sensitivity to WBMRI (18F-FDG PET/CT specificity = 86%, WBMR specificity = 43%, sensitivity = 75%). This study, however, was limited by the chosen clinical reference standard (disease activity defined as the presence of > 30% plasma cells at bone marrow aspiration of the iliac crest). Additionally, 18F-FDG PET/CT positivity was not defined in accordance with IMWG criteria (necessitating an osteolytic component) and number of focal lesions was not assessed. Similarly, in another prospective study, Mesguich et al. found that a greater number of lesions were detected on WBMRI than 18F-FDG PET/CT, but no diagnostic difference between modalities on a per patient basis [3]. However, MRI evaluation of diffuse disease with MY-RADS criteria [20] served as a reference standard for diffuse infiltration.

18F-FDG is the current recommended radiopharmaceutical for clinical imaging in myeloma, however, we found that the sensitivity of PET alone was only 54.3%. This is similar to the sensitivity of PET (59%) in Shortt et al. [16], where FDG-positive focal bone disease was defined as SUV_max_ > 2.5. The low sensitivity in the Shortt study was attributed to the fact that included patients were in different stages of treatment, but clearly there is additional contributing biology. One possible explanation for this is that there is reduced expression of the enzyme hexokinase-2 in individuals which has been reported in 11% of patients without 18F-FDG avid disease [21]. Hexokinase-2 is involved in the first step of glucose metabolism and is thought to be indicative of prolonged time to next treatment and progression-free survival [22]. Low-volume bone marrow plasma cell infiltration has also been suggested as a possible explanation for 18F-FDG negative disease [23].

The prognostic value of 18F-FDG PET/CT in myeloma has been clearly established. In a study by Zagmani et al., both progression-free and overall survival were adversely affected by the presence of extramedullary disease, three or more focal lesions at baseline and SUVmax greater than 4.2 [24]. In our study, there was a weak positive correlation between background bone marrow 18F-FDG avidity (SUVmax) and bone marrow plasma cell percentage, and sensitivity of bone marrow SUVmax:hepatic SUVmax >1 of 36.4% for detection of bone marrow infiltration. A recognised confounding factor in subjective assessment of background bone marrow infiltration on 18F-FDG PET/CT is that generalised increased bone marrow avidity may be observed in both plasma cell infiltration and in reactive marrow, e.g. secondary to anaemia, a common finding in myeloma patients.

In the diagnostic setting, it would appear that WBMRI and 18F-FDG PET/CT provide complementary information, and prospective studies exploring the diagnostic performance of hybrid techniques such as PET/MRI are needed. There has been very limited research exploring use of PET/MRI in myeloma to date. One previous study (*n* = 30) comparing 18F-FDG PET/CT and 18F-FDG PET/MRI in newly diagnosed myeloma concluded that 18F-FDG PET/MRI was a feasible imaging technique with comparable sensitivity to 18F-FDG PET/CT for focal bone lesion detection. However, there were statistically significant differences between 18F-FDG PET/CT and 18F-FDG PET/MR-derived SUVmax and SUVaverage with higher reported values for 18F-FDG PET/CT compared with 18F-FDG PET/MRI [25].

Limitations of our study include its retrospective nature and the lack of a pathological reference standard for every bone lesion; however, this limitation was partially offset by use of an independent clinical reference standard. Clinical factors such as patient co-morbidities/performance status were not accounted for, which would influence treatment decisions in day-to-day practice. The virtual management decision regarding treatment vs. surveillance was binary and did not address individual treatment regimens as this was not deemed pertinent to the study objectives. The small number of patients overall as well as with smouldering myeloma also limits the conclusions that can be drawn.

## Conclusion

WBMRI detects skeletal disease in a higher number of treatment naïve patients than 18F-FDG PET/CT and also detects a higher number of lesions per patient with concordance between 18F-FDG PET/CT and WBMRI in terms of disease positivity and lesion number in only 59% of patients. Imaging impacts on clinical management resulting in a significantly higher proportion of treated patients compared with clinical data alone. However, treatment decisions were not statistically different between modalities and either would be appropriate in initial staging, depending on local availability and expertise.

## Supplementary Information

ESM 1 (DOCX 16 kb)

## Data Availability

Data can be provided upon request to corresponding author.
