# Supplementary material for: Comparison of the diagnostic performance and impact on management of 18F-FDG PET/CT and whole-body MRI in multiple myeloma
Source: Eur J Nucl Med Mol Imaging. Author manuscript; Available in PMC 2021 Jul 1. (PMC8241666; doi:10.1007/s00259-020-05182-2)
Supplement: Supplementary Information [file EMS119963-supplement-Supplementary_Information.docx]

**Supplemental Table 1. MRI acquisition parameters**

| **Image contrast** | **T1-W** | **DWI*** | **T2-W** | **T1-W^+^** |
| --- | --- | --- | --- | --- |
| Sequence | DIXON 3D FLASH | DW-EPI | HASTE | DIXON 3D FLASH |
| Imaging plane | Axial | Axial | Axial | Coronal |
| Number of slices per imaging station | 40 | 40 | 40 | 128 |
| Acquired Slice Thickness  (mm) | 10 | 5 | 5 | 10 |
| Reconstructed Slice Thickness  (mm) | 5 | 5 | 5 | 2.0 |
| Slice gap  (mm) | 0 | 0 | 0 | 0 |
| FOV  (mm) | 500 | 500 | 500 | 500 |
| Acquired voxels  (mm x mm) | 2 x 1.6 | 3.9 x 3.9 | 2.0 x 2.0 | 1.9 x.17 |
| Reconstructed matrix | 640 | 256 | 512 | 288 |
| Reconstructed voxels (mm x mm) | 0.8 x 0.8 | 2.0 x 2.0 | 1.0 x 1.0 | 1.7 x 1.7 |
| Phase-encoding direction | AP | AP | AP | FH |
| TR  (ms) | 6.62 | 6270 | 400 | 6.76 |
| TE  (ms) | TE1 = 2.39,  TE 2 = 4.77 | 67 | 92 | TE1 = 2.39,  TE2 = 4.77 |
| Flip angle /  | 10 | 90 | 90; refocusing angle 180 | 10 |
| Number of signal averages | 1 | 2 (b50), 5 (b900) | 1 | 1 |
| Fat suppression | N/A | STIR | None | N/A |
| Acquisition Time  (per station) | 10s | 3min 22s | 16s | 20s |
| *b-values: 50 & 900 s/mm^2^; + pre and post gadolinium contrast agent | | | | |
